# B cells in the retinal pigment epithelium change with aging and IgM availability

**DOI:** 10.1016/j.isci.2026.117640

**Published:** 2026-09-26

**Authors:** Sarah E. Webster, Nicholas Deleon, Naomi L. Tsuji, Daken Heck, Michael J. Clemente, Nichol E. Holodick

**Affiliations:** 1 Center for Immunobiology, Department of Investigative Medicine, Western Michigan University Homer Stryker M.D. School of Medicine, Kalamazoo, MI 49007, USA

**Keywords:** B cells, aging, retina

## Abstract

Maintenance of the retina, the CNS tissue responsible for vision, is critical during aging. Historically considered immune privileged, the retina was thought to lack traditional immune components with resident glial cells maintaining tissue integrity. Emerging evidence suggests that immune suppression, rather than immune absence, governs retinal tolerance. Here, we identify B lymphocytes isolated from the retinal pigment epithelium (RPE), a CNS barrier niche, and show that their activation state and antibody repertoire are associated with secreted immunoglobulin M (IgM) availability and aging. In the absence of secreted IgM, B cells isolated from the RPE display a more activated phenotype and an altered B cell receptor repertoire, with age further modifying VH usage and CDR-H3 properties and expansion. Functionally, RPE B cells secrete antibodies following stimulation. These findings provide a foundation for understanding humoral regulation in CNS barrier tissues and mechanisms linking immune function to retinal aging and health.

## Highlights

• B cells are present within the health and aging retinal pigment epithelium (RPE)
• Age-associated differences are seen in the phenotype and repertoire of RPE B cells
• IgM-associated differences are seen in the phenotype and repertoire of RPE B cells
• RPE B cells secrete IgM following *ex vivo* stimulation

## Graphical abstract

**Figure:**
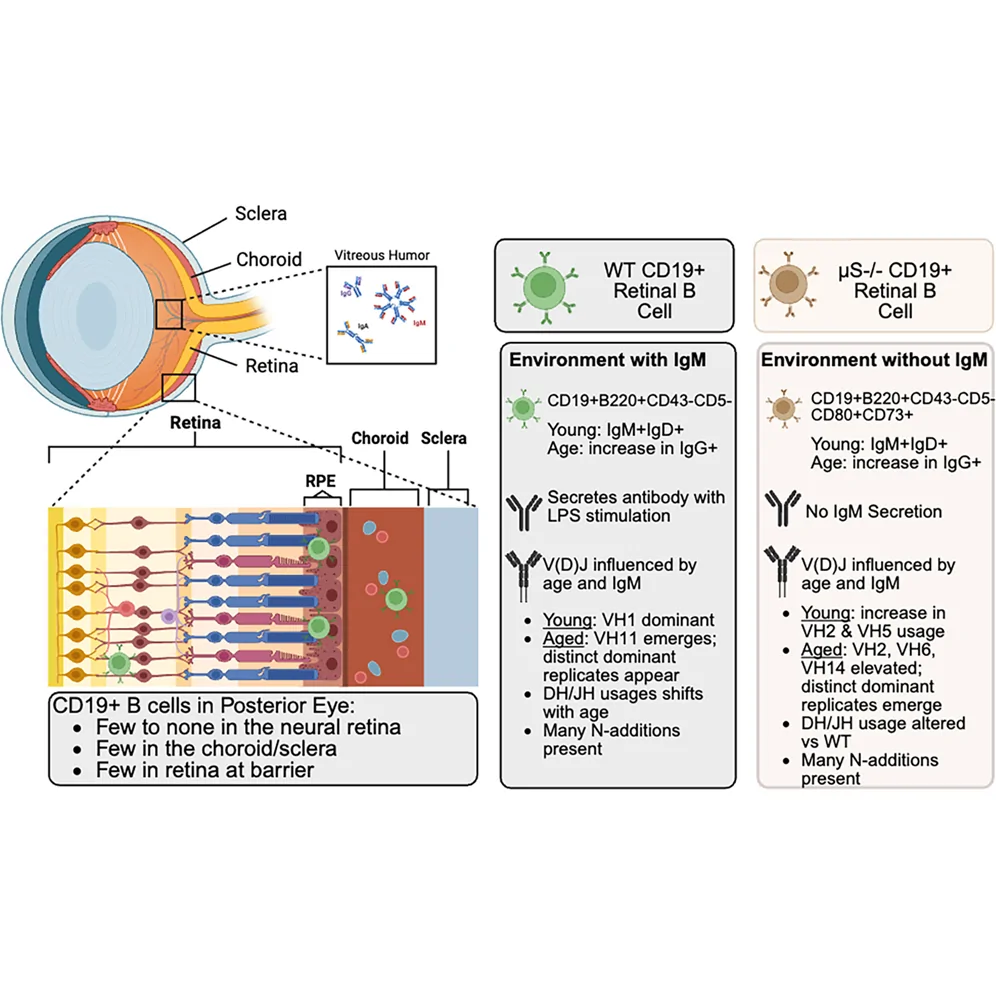

## Teaser

Immunology; Cell biology

## Introduction

Historically, the retina has been considered an immune-privileged site, protected from immune actions to preserve visual function. This concept of immune privilege was long interpreted as the absence of classical immune activity within the eye, with all immunological support provided by resident retinal glia. However, recent studies in the eye and central nervous system (CNS) indicate that immune privilege reflects not absence, but a controlled, actively regulated immune environment that enables selective interactions between ocular and systemic immune cells.^1^^,^^2^^,^^3^ Retinal immune responses have traditionally focused on microglia and, more recently, T cells.^4^^,^^5^^,^^6^^,^^7^ However, the presence and functional role of B cells in the ocular compartment remain poorly understood.

In the CNS, B cells are increasingly recognized as key players in immune regulation and pathology, with clear contributions to many CNS diseases such as multiple sclerosis^8^ and neuromyelitis optica.^9^ Yet, their presence under homeostatic conditions, particularly in the retina, has received little attention. Diverse subsets of murine B cells occupy canonical lymphoid and tissue-specific niches, each with distinct developmental and functional programs. B cells in the murine immune system comprise both conventional B2 cells, required for an adaptive response, and innate-like B-1 cells. Within the B2 compartment, where antigen-specific antibodies are produced, mature naive B2 cells are classified into two principal subsets: (1) follicular (FO) B cells which recirculate through lymph nodes and the spleen, serving as the primary mediators of T cell-dependent adaptive immunity,^10^ and (2) marginal zone (MZ) B cells, which reside in the splenic marginal zone and mount a more rapid response to antigen.^11^^,^^12^ Upon activation, FO B cells typically enter germinal centers where they undergo somatic hypermutation and selection before differentiating into long-lived memory B cells or antibody-secreting plasma cells. In contrast, MZ B cells respond with early IgM production and limited class switching, but only rarely participate in germinal center-dependent memory formation. Innate-like B-1 cells reside in serosal cavities, select mucosal and exocrine tissues, the spleen, and bone marrow.^13^ B-1 cells are capable of self-renewal and produce natural antibodies, which are largely non-specific, broadly cross-reactive, low-affinity, germline-like, present prior to antigen exposure, and change with age.^13^^,^^14^^,^^15^^,^^16^^,^^17^^,^^18^

Beyond classical niches, B cells also colonize tissue-specific environments. These include mucosal sites, like that in the colon^19^^,^^20^ and exocrine glands including the ocular lacrimal gland,^21^^,^^22^ and meningeal/CNS system border regions.^2^^,^^9^^,^^23^^,^^24^ At these tissue-specific sites, B cells contribute to local immunosurveillance, secrete antibodies, and modulate tissue-resident cells through myriad functions, including cytokine production, antigen presentation, and in some contexts, the expression of neuromodulators such as choline acetyltransferase.^25^ CD19 is a defining B-lineage marker expressed on all murine B cells except terminally differentiated plasma cells, which downregulate CD19. Recent studies reveal that the posterior eye is connected to a functional lymphatic circuit, supporting adaptive immune responses and suggesting a potential role of B cells in maintaining retinal homeostasis.^2^ Yet, whether B cells reside in the retina under normal, homeostatic conditions, especially during aging, remains unknown, leaving a critical gap in our understanding of retinal immune regulation.

Previously, we found that B cell-derived IgM plays a protective role in preserving retinal homeostasis during aging in mice.^26^ Here, we sought to determine whether B cells are present in the ocular compartment in aging mice. We found B cells in the retinal pigment epithelium (RPE) of both young and aged mice. We then investigated how changes in the ocular microenvironment, including loss of secreted IgM and aging, influence the phenotype, function, and repertoire of B cells isolated from the RPE. Using mice unable to secrete IgM (μs−/−) we found evidence of CD19^+^ B cells in the murine RPE in both young and aged mice, in both wild-type and knockout mice. These B cells isolated from the RPE were characterized by (1) phenotype, (2) ability to secrete antibodies upon stimulation, and (3) repertoire. Importantly, these studies demonstrate the presence of B cells associated with the RPE, a CNS barrier tissue, whose phenotype, activation state, and repertoire are associated with aging and IgM availability. We found that the absence of secreted IgM was associated with alterations in the phenotype and repertoire of B cells isolated from the RPE. Together, these data demonstrate that the presence of a small population of B cells isolated from the RPE across the lifespan, with phenotype and repertoire diversity associated with IgM availability, revealing a potential role for B lymphocytes in maintaining retinal homeostasis during aging.

## Results

### CD19^+^ B cells are found within the posterior eye in the retinal pigment epithelium

Recent evidence indicates that B cells might be involved in immune activity in the posterior eye in healthy mice.^2^ In addition, our previously published work demonstrated that B cell-derived IgM contributes to retinal homeostasis during aging, showing severe retinal degeneration in the absence of secreted IgM.^26^ To further examine the possibility that B cells are present within the eye, we analyzed a previously published bulk RNA-seq dataset from the neural retina (NCBI GEO: GSE156758)^27^ (neural retina defined in Figure S1A) for evidence of B lymphocytes (Figure S1B). Our analysis revealed low levels of CD19 expression and other B cell-related gene expression, supporting the possibility that B cells are present within ocular tissues under homeostatic conditions.

To directly assess their presence, we dissected three posterior eye compartments: (1) the neural retina, (2) retinal pigment epithelium (RPE), and (3) choroid/sclera (c/s). Each layer was carefully dissected, digested to a single-cell suspension; single-cell suspensions underwent positive selection for CD19^+^ cells through a magnetic column (Figure 1A). The CD19^+^ enriched cell suspensions were then sorted for further analyses. Examining these three locations in young C57BL/6 mice, we discovered that the primary location for live CD19^+^ cells was within the RPE layer of the retina (Figure 1B). The neural retina contained very few live CD19^+^ B cells (0.035%), whereas the RPE contained more live CD19^+^ B cells (1.8%), making the RPE the primary site of retinal B cells. Therefore, we focused on CD19^+^ B cells isolated from the RPE layer for further analyses.

**Figure 1:**
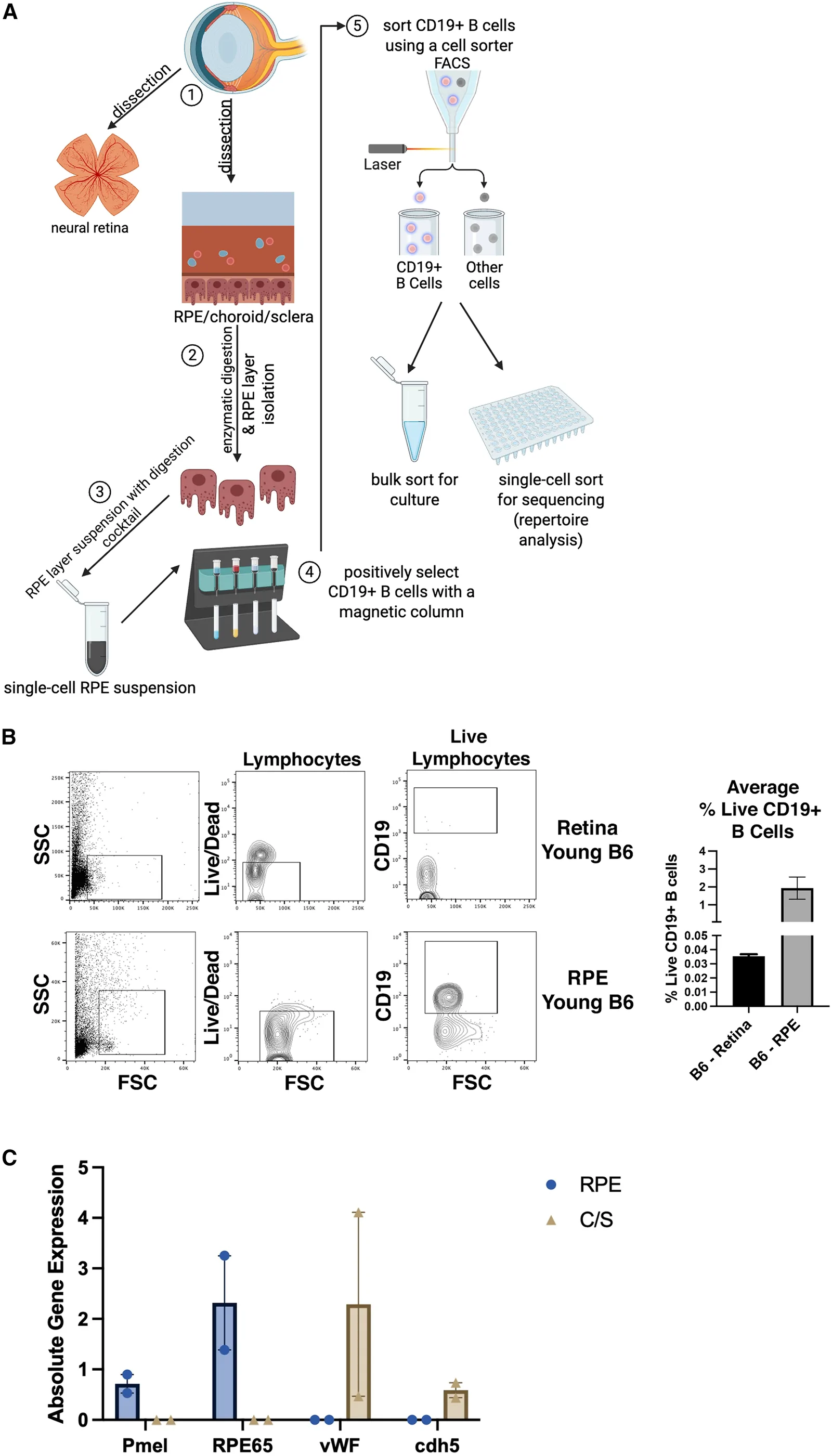
CD19^+^ B cells are found within the posterior eye in the retinal pigment epithelium (A) The diagrammed procedure for tissue processing in separation of the neural retina, RPE, and C/S followed by CD19 positive selection and FACS analysis. (B) Single cell suspensions from neural retina or RPE were made in young (3 months old) C57BL/6 mice. Flow plots show that many more cells are found in RPE as compared to neural retina, which was then quantified. Sample size: *n* = 3 per group. Data are represented as mean SEM. (C) Tissue dissections were done by gross morphology and anatomical landmarks and purity of dissection was confirmed by RT-qPCR for tissue-specific markers (RPE genes: Pmel, premelanosome protein; produces and stores pigment; RPE65, enzyme responsible for conversion of all-trans-retinol to 11-cis-retinol; C/S genes: vWF, von Willebrand factor expressed in blood vessels of choroid, specifically in endothelial cells; cdh5, cadherin 5 a cell-cell adhesion molecular found in vascular endothelium of the choroid). Sample size: *n* = 5 per group; repeated in duplicate. Data are represented as mean ± SEM.

Importantly, tissue identity (RPE versus c/s) was confirmed by gross histological analysis followed by RT-qPCR for canonical retinal/RPE and choroidal markers (Figure 1C). We observe absence of detectable endothelial markers (vWF, CDH5) within our isolated RPE samples, demonstrating unlikely vascular contamination. Conversely, choroid/sclera samples, which are highly vascular, show strong endothelial marker expression and no expression of RPE related genes (RPE65) (Figure 1C).

### CD19^+^ B cells isolated from the RPE secrete antibody upon stimulation

To determine whether B cells in the RPE can secrete antibody, we stimulated CD19^+^ B cells isolated from the RPE of young and aged wild type (WT) and μs−/− mice (lacking secreted IgM) with lipopolysaccharide (LPS). RPE tissue was dissected, underwent enzymatic digestion, and then CD19^+^ cells were isolated by positive selection and sort purification (Figure 2A). Representative flow plots are shown in Figure 2B. The sorted CD19^+^ cells were then cultured with LPS, a component of the outer membrane of gram-negative bacteria, which binds to toll-like receptor 4 (TLR4) on B cells, inducing T cell-independent B cell activation and antibody secretion.

**Figure 2:**
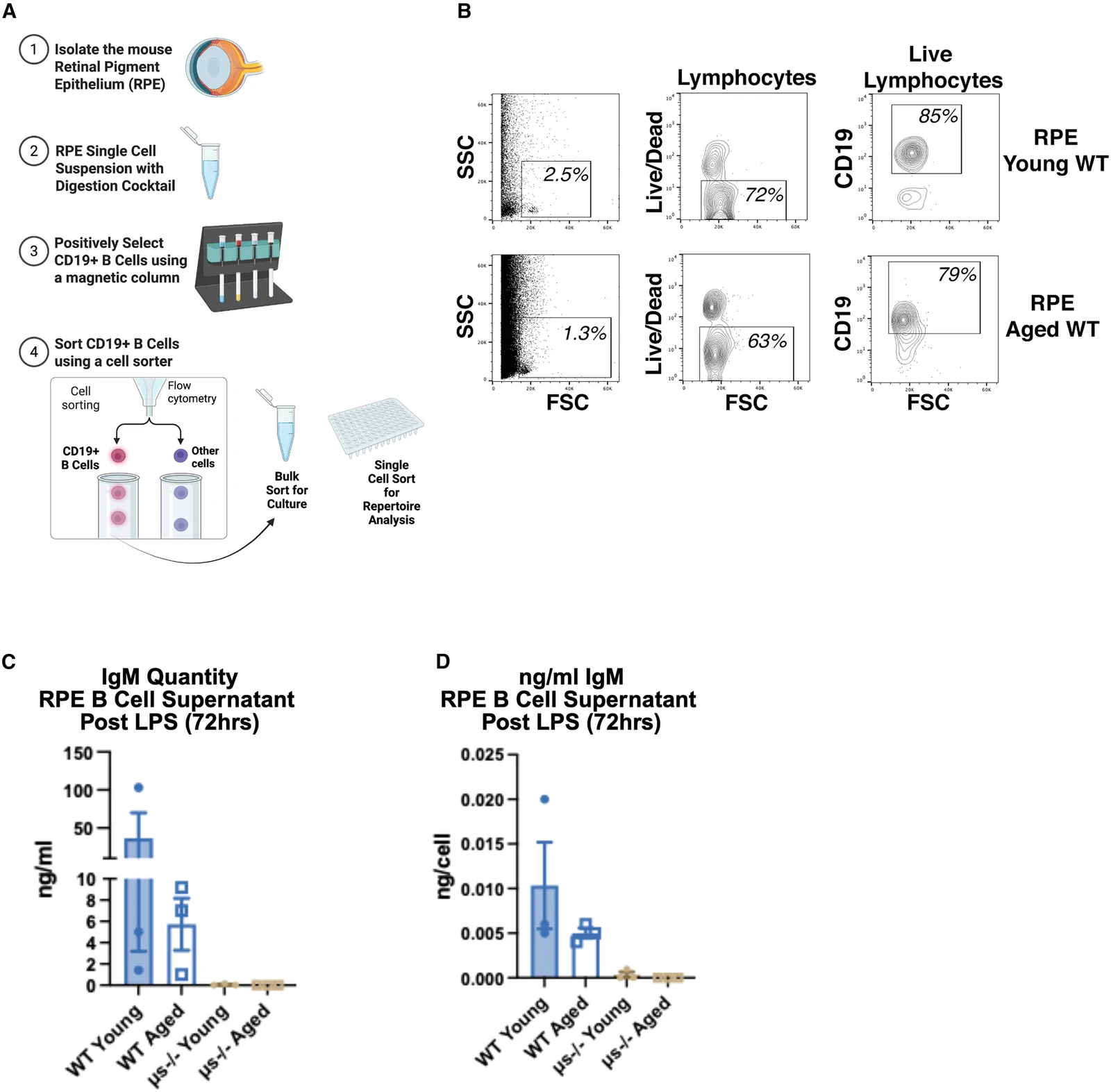
CD19^+^ B cells found in the RPE respond to LPS (A) Murine RPE was isolated from young (3–4 months) and aged (19–26 months) WT and μs−/− mice from both male and female mice. The RPE was digested, prepared as a single cell suspension, and then selected for CD19^+^ B cells using magnetic beads. CD19^+^ B cells obtained from the RPE were then stained to determine live cells and CD19^+^ B cells. (B) Representative FACS plots display the gating strategy for sorting live CD19^+^ B cells. (C and D) B cells from the RPE were isolated as described in (A) and were then bulk sorted. The cells were placed into culture media with 25 μg/ml of LPS for 72 h and then supernatant was collected. IgM was quantified by ELISA. (C) The amount (ng/mL of IgM) in the supernatant of cultured RPE B cells. (D) The ng/cell of IgM produced from cultured RPE B cells. Results are based on 3 independent experiments; each dot represents an independent experiment of 3–6 pooled mice (6–12 pool eyes) per experiment. The total number of mice used in the 3 independent experiments are as follows: *n* = 20 3-month-old WT mice (*n* = 40 eyes), *n* = 22 19–26-month-old WT mice (*n* = 44 eyes), *n* = 18 3-month-old mice μs−/− mice (*n* = 36 eyes), *n* = 16 20–26-month-old μs−/− mice (*n* = 32 eyes). Data are represented as mean ± SEM. Statistics used: Sharpiro-Wilk test for normality and Mann-Whitney test.

Supernatants from LPS-stimulated CD19^+^ B cells from the RPE were assessed for IgM production using ELISA. B cells from young WT mice produced an average of 54 ng/mL IgM, whereas aged WT B cells produced 4 ng/mL (Figure 2C). As expected, B cells from μs−/− mice did not secrete IgM (Figures 2C and 2D), consistent with their deletion of secretory IgM. When normalized per cell, young WT CD19^+^ B cells generated approximately 0.013 ng/cell IgM compared to 0.006 ng/cell from aged WT CD19^+^ B cells (Figure 2D). These data demonstrate that B cells isolated from the RPE respond to LPS stimulation and secrete antibody.

### CD19^+^ B cells isolated from the RPE have a repertoire shaped by age and secreted IgM

Given the nature of the retinal B cell niche, including tight physical barriers, low antigen load, and strong influence of aging on the environment, the B cells that persist here likely undergo selection pressures markedly different from those experienced in peripheral lymphoid tissue. Thus, defining how age and IgM status shape the repertoire of B cells isolated from the RPE provides a critical window for understanding how antibody potential is sculpted within the retina barrier environment.

IgM participates broadly in regulating immune homeostasis, shaping early B cell selection, and modulating tissue-level immunological tone.^28^^,^^29^^,^^30^^,^^31^^,^^32^^,^^33^^,^^34^^,^^35^^,^^36^^,^^37^^,^^38^^,^^39^^,^^40^ However, how the presence or absence of secreted IgM influences the B cell repertoire within specialized immune environments, such as the retina, has not been defined, especially in the context of aging. As we have previously demonstrated, lack of secreted IgM leads to retinal degeneration in aged mice.^26^ To determine how aging and secreted IgM deficiency shape antibody repertoire in CD19^+^ B cells isolated from the RPE, we conducted single-cell V(D)J sequencing on CD19^+^ B cells isolated from the RPE of both young and aged μs−/− mice. This allowed us to resolve VH, DH, and JH segment usage on a per-cell basis and evaluate how age and presence of IgM influenced the RPE B cell antibody repertoire (Figure 3).

**Figure 3:**
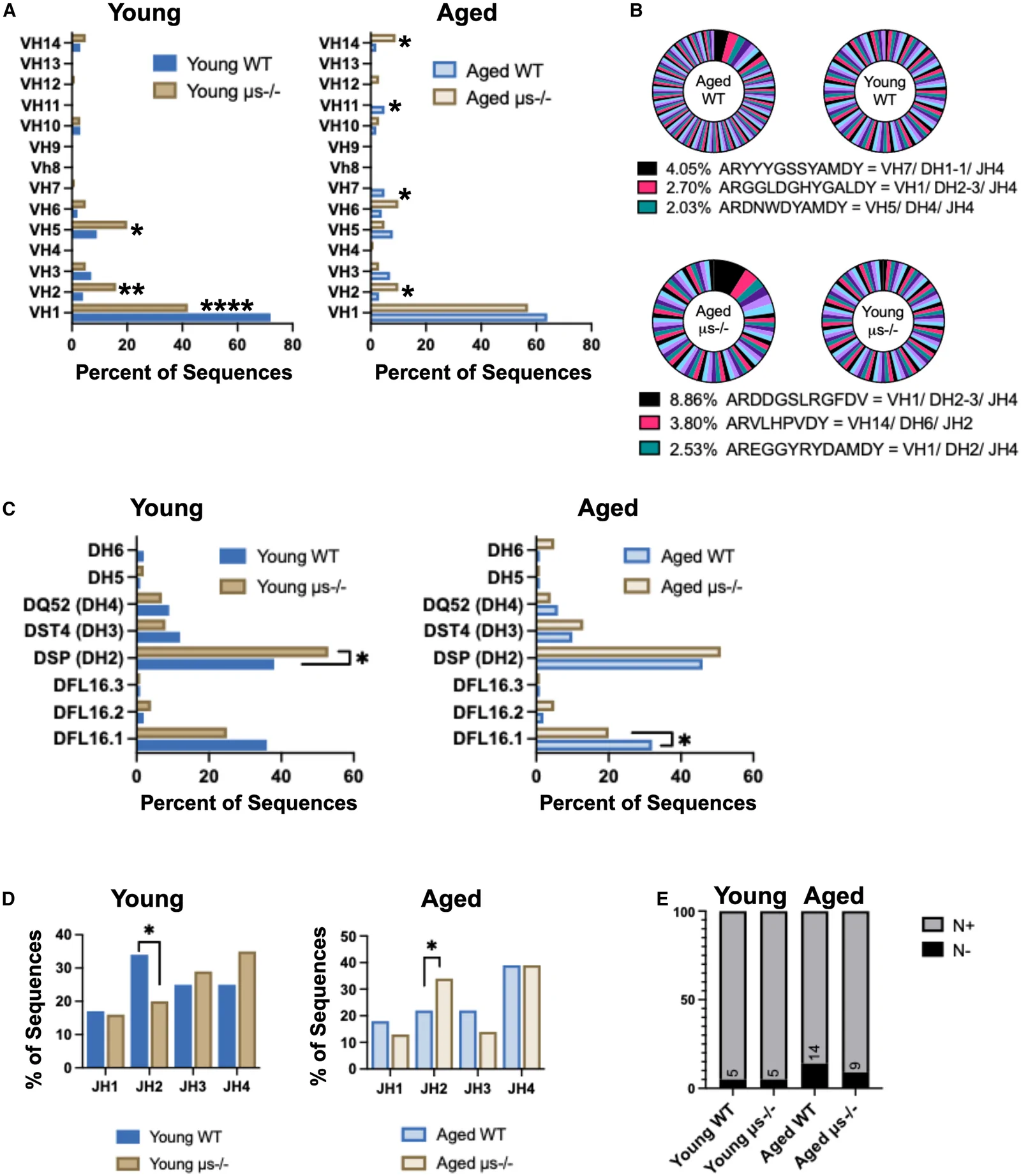
Repertoire analysis of CD19^+^ RPE B cells Murine RPE was isolated from young (3–4 months) and aged (23–26 months) WT and μs−/− mice from both male and female mice. The RPE was digested, prepared as a single cell suspension, and then selected for CD19^+^ B cells using magnetic beads. CD19^+^ B cells obtained from the RPE, were stained to determine live cells and CD19^+^ B cells (as shown in Figure 3A) and were then single-cell sorted into a 96-well plate with cell lysis buffer. The VH region was amplified and sequenced as detailed in Materials and Methods. (A) The percent of V_H_ gene segment usage. (B) Distribution of replicate CDR-H3 sequences (sequences with the same CDR-H3 sequence). Each color represents a unique CDR-H3 amino acid sequence. (C) The percent of D_H_ gene segment usage. (D) The percent of J_H_ gene segment usage. (E) The percent of sequences containing zero N-additions (black bars) or 1 or more N-additions (light gray bars) at both junctions is shown with replicate sequences included in the analysis. Results are based on 4 independent experiments with sequences combined from each independent experiment (*n* = 15 3-month-old mice WT mice (*n* = 30 eyes), *n* = 18 23–26-month-old WT mice (*n* = 36 eyes), *n* = 12 3-month-old μs−/− mice (*n* = 24 eyes), *n* = 10 23–26-month-old μs−/− mice (*n* = 20 eyes). Data are represented as mean ± SEM. Statistics used: 2×2 chi-square test. Asterisks for *p* values: ∗*p* < 0.05, ∗∗*p* < 0.01, ∗∗∗*p* < 0.001, ∗∗∗∗*p* < 0.0001.

Analysis of VH segment usage revealed clear age- and secreted IgM-dependent differences in repertoire selection. In young WT mice, retinal B cells showed a strong predominance of VH1 usage (72% vs. 42% in young μs−/−; *p* < 0.001, ×2, 2 × 2), suggesting that the unperturbed retinal microenvironment favors a relatively focused VH bias in early life. Young μs−/− retinal B cells, however, exhibited increased representation of VH2 (16% vs. 4% in young WT; *p* < 0.05) and VH5 (20% vs. 9% in young WT; *p* < 0.01) (Figure 3A), indicating that even in young mice, the absence of secreted IgM shifts VH usage. With age, these patterns diverged further. Although substantial VH1 usage persisted in aged WT and μs−/− B cells (64% aged WT vs. 57% aged μs−/−), aged WT mice showed a relative increase in VH11 usage that was not observed in aged μs−/− mice (5% vs. 0%; *p* < 0.05). Aged μs−/− retinal B cells demonstrated elevated usage of VH2 (10% vs. 3% in aged WT; *p* < 0.05), VH6 (10% vs. 4% in aged WT; *p* < 0.05), and VH14 (9% vs. 2% in aged WT; *p* < 0.05) (Figure 3A). These results suggest that both aging and secreted IgM deficiency alter the VH landscape of retinal B cells, which may have implications for responses to changes in antigen exposure, niche-dependent selection pressures, or the inflammatory environment of the μs−/− and aging retina.

Expansion of repetitive CDR-H3 regions exhibited similar age- and secreted IgM deficient-dependent divergence. Young mice displayed highly variable and largely unexpanded repertoires, consistent with limited antigenic stimulation in the healthy young retina (Figure 3B). However, overuse of particular CDR-H3 regions became more pronounced with age (Figure 3B). The most CDR-H3 in aged WT mice was ARYYGSSYAMDY [VH7/DH1-1/JH4] (4.05%), whereas aged μs−/− mice were dominated by a distinct and more heavily expanded CDR-H3, ARDDGSLRGFDV [VH1/DH2-3/JH4] (8.86%) (Figure 3B). The emergence of unique dominant CDR-H3 regions in aged WT vs. aged μs−/− mice is consistent with differences in the expansion of specific CDR-H3 configurations associated with IgM availability during aging in the RPE.

DH and JH gene usage patterns further demonstrated that repertoires are influenced by aging and absence of secreted IgM. Young μs−/− retinal B cells used the DSP(DH2) gene segment at significantly higher frequencies than their WT counterparts (53% vs. 38% WT; *p* < 0.05) (Figure 2C), suggesting that early-life DH selection is sensitive to IgM status. During aging, WT mice favored DFL16.1 to a greater extent than aged μs−/− mice (32% vs. 20% aged μs−/−; *p* < 0.05) indicating that age and IgM influence DH selection (Figure 3C). JH usage also diverged with age and the lack of secreted IgM in the environment. Young WT retinal B cells preferentially utilized JH2 over young μs−/− mice (34% vs. 20% young μs−/−; *p* < 0.05), but this trend was reversed with age; in aged WT mice, we observed a decrease in JH2 usage as compared to aged μs−/− mice (22% vs. 34% aged μs−/−; *p* < 0.05) (Figure 3D). These data suggest that both age and availability of secreted IgM may alter the selection of particular JH segments.

Analysis of N-additions (Figure 3E) suggest that the CD19^+^ B cells isolated from the RPE predominantly consisted of postnatally derived cells. Most sequences contained N-additions, consistent with TdT activity during postnatal B cell development, whereas fetal-origin-like sequences lacking N-additions were rare in young WT (5/100 sequences) and young μs−/− (5/100 sequences) mice. Interestingly, aging showed a small increase in retinal B cells that lack N-additions (aged WT, 14/100 sequences; and aged μs−/−, 9/100 sequences); however, this was not a significant difference (Figure 3E). These data suggest a model in which CD19^+^ B cells isolated from the RPE are derived from postnatal sources, with minimal contributions from fetal-derived B cell lineages; however, further experimentation is needed to confirm such a model.

### The hydrophobicity and amino acid composition of the CDR-H3 loop of CD19^+^ B cells isolated from the RPE are impacted by age and secreted IgM

Among the six complementary determining regions (CDRs) of antibodies, CDR-H3 is the main site of antigen binding. Hydrophobic CDR-H3 loops have been shown to be essential for certain broadly neutralizing antibodies against HIV,^41^ whereas antibodies with highly charged CDR-H3 loops are often autoreactive.^42^^,^^43^ We evaluated the average hydrophobicity of each CDR-H3 loop using the Kyte-Doolittle scale. No changes in the hydrophobicity of the CDR-H3 loop were observed in the young mice. In contrast, in the aged, the CDR-H3 loop of retinal B cell IgM decreased in charge (increased in hydrophobicity) in the absence of IgM (−0.262 ± 0.021 in aged WT vs. −0.041 ± 0.039 in aged μs−/−, *p* < 0.0001) (Figure 4A). DH use has been shown to be primarily responsible for changes in hydrophobicity, with DH length positively correlating with the increased hydrophobicity of CDR-H3.^44^ We observed that the hydrophobicity of the aged WT RPE B cell CDR-H3 loops was positively correlated with the length of the respective DH genes (Figure 4B).

**Figure 4:**
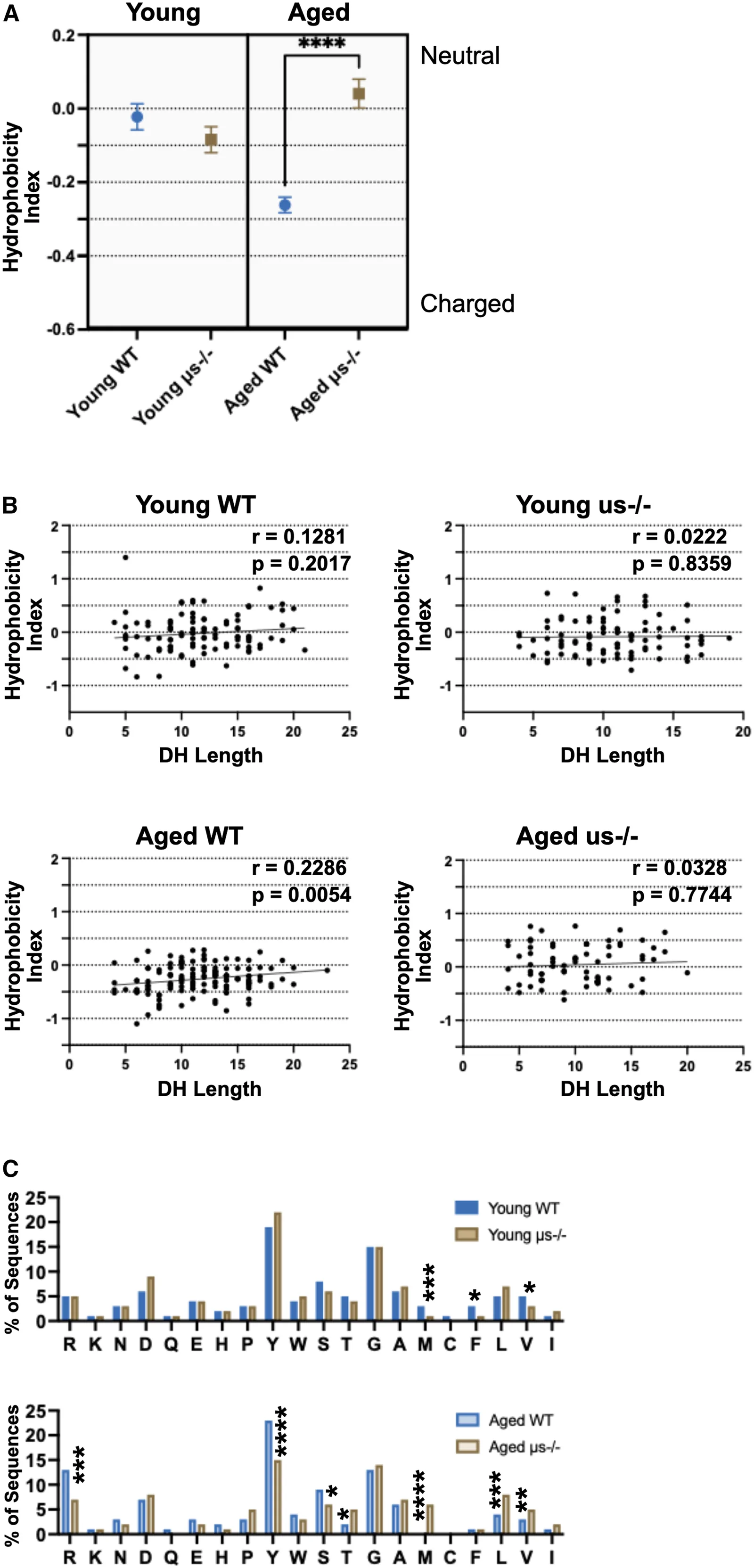
Hydrophobicity and amino acid composition of the CDR-H3 loop of CD19^+^ B cells isolated from the RPE CD19+ B cells obtained from the RPE, were stained to determine live cells and CD19^+^ B cells (as shown in Figure 3A) and were then single-cell sorted into a 96-well plate with cell lysis buffer as indicated in Figure A. The VH region was amplified and sequenced as detailed in Materials and Methods. (A) The average charge or hydrophobicity index of the CDR-H3 loop region of IgM from CD19^+^ RPE B cells. (B) Correlation of average charge of the CDR-H3 loop region with DH length. (C) The percent of each amino acid used within the CDR-H3 was determined for RPE B cells as indicated. Results are based on sequences obtained from experiments performed in Figure 4 (see Figure 3 for number of animals and independent experiment number). Data are represented as mean ± SEM. Statistics used: Mann-Whitney test for (A), Spearman’s rank correlation coefficient for (B), and Chi-square for (C). Asterisks for *p* values: ∗*p* < 0.05, ∗∗*p* < 0.01, ∗∗∗*p* < 0.001, ∗∗∗∗*p* < 0.0001.

The hydrophobicity of CDR-H3 is influenced by DH use, JH use, N-additions, and/or amino acid content.^43^ Mature murine B cells display a predominance of tyrosine and glycine within the CDR-H3 region,^45^^,^^46^ and changes in the amino acid content within this region can result in decreased B cell development, decreased antibody production, and increased infection susceptibility.^47^^,^^48^^,^^49^ Interestingly, we found specific differences in the amino acid content within the CDR-H3 region of RPE B cell IgM between WT and μs−/− mice (Figure 4C). In young mice, we observed a significant decrease in the use of methionine (3% vs. 1%), phenylalanine (3% vs. 1%), and valine (5% vs. 3%). More differences were observed in aged RPE B cells. Interestingly, there was a decrease in tyrosine (23% vs. 15%), serine (9% vs. 6%), and arginine (13% vs. 7%) usage in aged μs−/− mice compared to that in aged WT mice. Aged μs−/− mice also showed an increase in threonine (2% vs. 5%), methionine (0% vs. 6%), leucine (4% vs. 8%), and valine (1% vs. 2%) usage compared to aged WT mice. These results reveal that both age and presence of secreted IgM impact the hydrophobicity and amino acid content of the CDR-H3 loop in RPE B cell populations.

Together, these findings show that aging and secreted IgM deficiency are associated with the differences in the molecular composition, clonal expansion, and structural properties of the RPE B cell repertoire. Both the increased hydrophobicity and altered amino acid usage in the μs−/− mice highlight an association between IgM availability, B cell diversity, and antigen-binding architectures within the retinal niche during aging.

### Surface phenotype of CD19^+^ B cells within the RPE

To gain insight into the identity of CD19^+^ B cells within the RPE under homeostatic conditions, we performed immunophenotyping. The RPE layer was isolated from young (3–4 months) and aged (18–24 months) WT and μs−/− mice of both sexes and stained for comparative analysis (Figure 1A, cells were stained for phenotypic analysis after digestion in step 3). No sex-associated differences were observed for the outcomes examined; therefore, data from male and female mice were combined for the analyses presented. The representative gating strategy is shown in Figure 5A. Gating on live lymphocytes, we observed no significant difference in the percentage of live lymphocytes between young (WT: 5.4% ± 1.5% vs. μs−/−: 4.6% ± 1.2%) or aged (WT: 2.5% ± 0.5%; μs−/− 2.4% ± 0.5%) WT and μs−/− mice (Figure 5B). Furthermore, upon examination of B220+CD5^−^ live lymphocytes, we found that their frequency was consistent across all ages and backgrounds (young WT: 3.7% ± 1.4%; aged WT: 1.8% ± 0.6%; young μs−/−: 2.7% ± 0.5%; aged μs−/− 2.2% ± 0.8%) (Figure 5C). Gating on B220+CD5^−^ cells, we examined CD19 and CD43 expression (Figure 5A). We found that the overwhelming majority of B220+CD5^−^ B cells were CD19^+^ and CD43^−^ (Figure 5D). The number of B220+CD19^+^CD5^−^CD43^−^ B cells within the RPE of young WT and μs−/− mice was higher, although not significantly higher, than the number observed in aged WT and μs−/− mice (young WT: 976 ± 620; aged WT: 271 ± 118; young μs−/−: 1441 ± 797; aged μs−/− 345 ± 188) (Figure 5E). It should be noted that each experiment was performed by pooling an average of 10 mice (20 eyes) per experiment; therefore, these calculated numbers of B cells within the RPE are not per eye.

**Figure 5:**
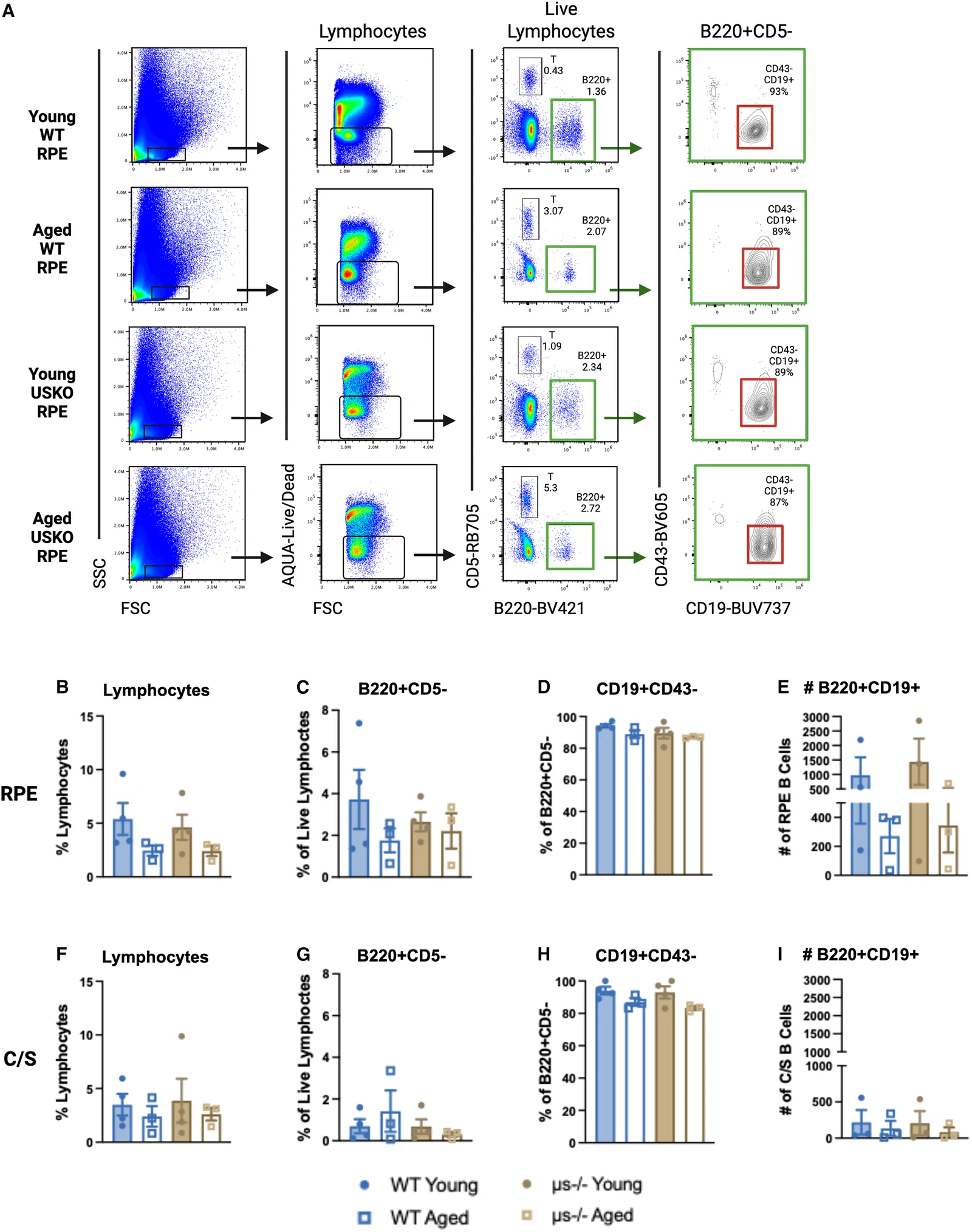
Surface phenotype of CD19^+^ B cells found in the RPE layer of the eye Murine RPE was isolated from young (3–4 months) and aged (22–24 months) WT and μs−/− mice from both male and female mice. The RPE was digested, prepared as a single cell suspension, and then stained for phenotype analysis. (A) Representative flow plots of B cell phenotyping from isolated RPE. (B) The percent of lymphocytes found in the RPE. (C) The percent of live lymphocytes in the RPE that are B220+CD5^−^. (D) The percent of live B220+CD5^−^ lymphocytes in the RPE that are CD19^+^CD43^−^. (E) The number of B220+CD19^+^CD5^−^CD43^−^ B cells found in the RPE. Representative flow plots for the choroid/sclera (C/S) are shown in Figure S3. (F) The percent of lymphocytes found in the C/S. (G) The percent of live lymphocytes in the C/S that are B220+CD5^−^. (H) The percent of live B220+CD5^−^lymphocytes in the C/S that are CD19^+^CD43^−^. (I) The number of B220+CD19^+^CD5^−^CD43^−^ B cells found in the C/S. Results shown are an average of 3 independent experiments for young mice (individual animals pooled in each of 3 experiments with 3–6 mice per experiment) WT: *n* = 17 mice/34 eyes, μs−/−: *n* = 13 mice/26 eyes; and 3 independent experiments for aged mice (individual animals pooled in each of 3 experiments) WT: *n* = 13 mice/26 eyes, μs−/−: *n* = 18 mice/36 eyes. Data are represented as mean ± SEM. Statistics used: Sharpiro-Wilk test for normality and Mann-Whitney test.

Next, we examined the frequency of live lymphocytes that were B220+CD5^+^ (representative gating strategy shown in Figure S2A). We observed a low frequency of B220+CD5^+^ cells in all mice (young WT: 0.019% ± 0.001%; aged WT: 0.191% ± 0.180%; young μs−/−: 0.021% ± 0.010%; aged μs−/− 0.132% ± 0.084%) (Figure S2B). The percent of B220+CD5^+^ cells that were CD19^+^CD43^+^ varied by aged and presence of secreted IgM, but no significant differences were observed (Figure S2C). The number of B220+CD5^+^CD19^+^CD43^+^ B cells within the RPE (young WT: 5.0 ± 4.0; aged WT: 4.0 ± 2.0; young μs−/−: 1.7 ± 1.2; aged μs−/− 7.7 ± 6.7) (Figure S2D) was significantly lower than the number of B220+CD19^+^CD5^−^CD43^−^observed in the RPE (345–1441 cells, Figure 5E). We also observed a small T cell (CD5+B220-) population within the RPE and these increased with age, although not significantly (WT young: 1.1% ± 0.5% vs. WT aged: 6.1% ± 4.0%; μs−/− young: 1.3% ± 0.2% vs. μs−/− aged: 9.0% ± 4.9%) (Figure S2E). No significant differences were observed in the number of B220-CD5^+^ T cells within the RPE (young WT: 1202 ± 995.3; aged WT: 575.3 ± 181.3; young μs−/−: 2134 ± 1280; aged μs−/− 1200 ± 465.5) (Figure S2F).

Since the CD19+B220+CD5^−^CD43^−^ B cells were the predominate B cells found within the RPE we examined whether these were also present in the C/S fraction (Figures 5F–5I and S3). The representative gating strategy is shown in Figure S3. However, their abundance in the C/S was markedly lower than that in the RPE layer (Figure 5E compared to Figure 5I; 976 ± 620 cells in the young WT and 1441 ± 797 cells in the young μs−/− RPE compared to 219 ± 170 cells in young WT and 207 ± 166 in the young μs−/− C/S tissue). The same low abundance of these B cells in the C/S compared to RPE was also observed in the aged mice: 271 ± 118 cells in the aged WT and 345 ± 188 cells in the aged μs−/− RPE compared to 135 ± 103 cells in aged WT and 85 ± 63 in the aged μs−/− C/S tissue (Figure 5E compared to Figure 5I).

These results demonstrate that most of the B cells found within the RPE have the phenotype of B220+CD19^+^CD5^−^CD43^−^, which is most consistent with that of B2 cells. Given that B2 cells have been identified in CNS-adjacent tissues,^9^^,^^50^ the phenotype of the B cells isolated from the RPE is most consistent with a B2-like subset.

### B220+CD19^+^CD5^−^CD43^−^ B cells found in the RPE are IgM+IgD+ and upregulate CD80 and CD73 in the absence of secreted IgM

To further explore the phenotype of the B220+CD19^+^CD5^−^CD43^−^ B cells in the RPE, we performed a comprehensive phenotypic analysis of these B cells using canonical markers of B cell developmental state, activation, and regulatory potential. We found that the majority of CD19+B220+CD43^−^ B cells were IgM+IgD+ (Figures 6A and 6A′). Young WT RPE B cells were found to be 87.0% ± 3.61% IgM+IgD + while 85.6% ± 5.24% are IgM+IgD+ in aged WT RPE B cells. In μs−/− mice, both young and aged mice have high levels of IgM+IgD + RPE B cells (72.2% ± 4.93% and 77.7% ± 4.70%, respectively), but like in aging WT mice, IgG+ RPE B cells are observed (Figures 6B and 6B′), while in young WT we see little to no IgG+ RPE B cells (0.26% ± 0.17% young WT vs. 11.1 ± 5.22 aged WT vs. 6.16% ± 5.69% young μs−/− vs. 5.04 %± 3.65% aged μs−/−). The reduction in IgM+IgD + B cells with the increase in IgG+ in aged and μs−/− mice could reflect either local class-switch recombination, recruitment of peripherally switched B cells, or altered selection of the existing subsets. Similar IgM+ to IgG+ composition shifts have been described in other B cell/CNS border regions with age.^50^^,^^51^^,^^52^

**Figure 6:**
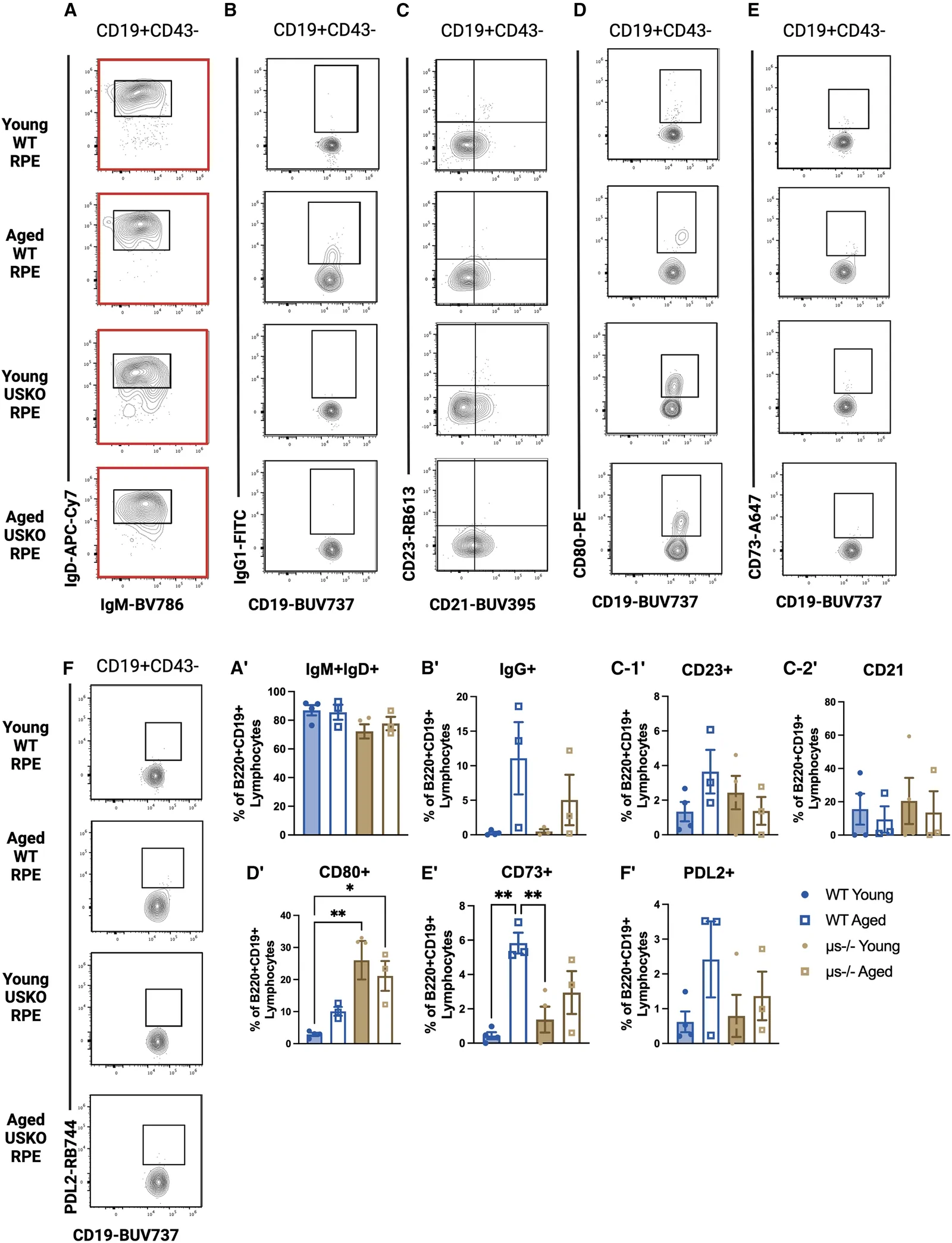
B cells found in the RPE are IgM+IgD + B cells that increase CD80 expression in the absence of secreted IgM Murine RPE was isolated from young (3–4 months) and aged (22–24 months) WT and μs−/− mice from both male and female mice. The RPE was digested, prepared as a single cell suspension, and then stained for phenotype analysis. Live B220+CD5^−^ lymphocytes that are CD19^+^CD43^−^were analyzed for (A and A′) IgM/IgD expression, (B and B′) IgG expression, (C, C-1′, and C-2′) CD23 and CD21 expression, (D and D′) CD80 expression (E and E′) CD73 expression, and (F and F′) PD-L2 expression. Results shown are an average of 3 independent experiments for young mice (individual animals pooled in each of 3 experiments with 3–6 mice per experiment) WT: *n* = 17 mice/34 eyes, μs−/−: *n* = 13 mice/26 eyes; and 3 independent experiments for aged mice (individual animals pooled in each of 3 experiments) WT: *n* = 13 mice/26 eyes, μs−/−: *n* = 18 mice/36 eyes. Data are represented as mean ± SEM. Statistics used: Shapiro-Wilk test for normality and Mann-Whitney test. Asterisks for *p* values: ∗*p* < 0.05, ∗∗*p* < 0.01, ∗∗∗*p* < 0.001, ∗∗∗∗*p* < 0.0001.

We found that few B220+CD19^+^CD5^−^CD43^−^lymphocytes expressed CD23, CD21, and PDL2; however, neither age nor genetic background significantly impacted the expression levels (Figures 6C, 6F, 6C-1′ and 6C-2′). We found that CD80, a marker for B cell activation, was significantly increased in the μs−/− mice compared to the age-matched counterpart (young WT: 2.89% ± 0.51% vs. young μs−/−: 26.0 ± 6.02; *p* = 0.0071) (Figure 6D’). Additionally, CD73, a marker associated with regulatory function, increased in aged WT mice compared to that in young WT mice (aged WT: 5.83% ± 0.61% vs. young WT: 0.44% ± 0.20%; *p* = 0.0021) (Figure 6E’). These data suggest that the lack of secreted IgM is associated with a more activated RPE B cell phenotype.

Next, we compared the same phenotypic profiling of B cells found in the RPE to circulating peripheral B cells. Representative gating of circulating peripheral B cells is displayed in Figure S4A. We observed a significantly higher average percent of circulating live lymphocytes (young WT: 61% ± 3.4%; young μs−/−: 61% ± 2.5%; Figure S4B) than in the young RPE (about 5%; Figure 5B). In addition, a higher average percent of circulating B220+CD5^−^live lymphocytes (young WT: 63% ± 1.6%; young μs−/−: 59% ± 2.3%; aged WT: 70% ± 2.9%; aged μs−/−: 72% ± 3.4%; Figure S4C) than in the young RPE (2.6%–3.7%; Figure 5C). These circulating B220+CD5^−^live lymphocyte were 94%–98% CD19^+^CD43^+^ (Figure S4D). On average 84% of young WT, 72% of young μs−/−, 74% aged WT, and 68% aged μs−/− circulating B220+CD19^+^CD5^−^CD43^−^ B cells are CD23^+^CD21^+^ (Figure S4E), which is unlike the B220+CD19^+^CD5^−^CD43^−^ B cells found in the RPE that express little CD23 and moderate CD21 (Figures 6C, 6C-1′, and 6C-2′). Furthermore, there are significantly more B cells that are phenotypically CD5^+^ B-1a cells (B220loCD5+CD43^+^CD19^+^CD23^−^) in circulation (young WT: 1.1% ± 0.2%; young μs−/−: 2.7% ± 0.3%; aged WT: 0.7% ± 0.1%; aged μs−/−: 1.3% ± 0.2%; Figure S4F) than observed in the RPE (young WT: 0.019% ± 0.001%; young μs−/−: 0.021% ± 0.010%; Supplement Figure S2B). We also observed a higher fraction of B220-CD5^+^ T cells within circulation (young WT: 15.3% ± 0.32%; young μs−/−: 14.5% ± 0.73%; aged WT: 12.7% ± 1.14%; aged μs−/−: 11.3% ± 0.93%; Figure S4H) than in the RPE WT: 1202 ± 995.3; young μs−/−: 2134 ± 1280; Figure S2F). These results demonstrate the composition of B cells and T cells found in the RPE is significantly different than that of peripheral circulation.

These results identify a population of B220+CD19^+^CD5^−^CD43-IgM+IgD+CD23lo/-CD21lo B cells isolated from the RPE that is approximately 6-fold more abundant in the RPE than in the choroid/sclera fraction in young mice and approximately 3-fold more abundant in the RPE than in the choroid/sclera in aged mice.

## Discussion

Barrier tissues, such as the meninges, are home to specialized immune populations, including tissue-resident B cells, which are thought to contribute to local immune surveillance and maintain homeostasis.^50^^,^^51^ Our previous work demonstrated lack of secreted IgM alone caused severe retinal degeneration in old age.^26^ Herein, we identify CD19^+^ B cells within the retinal pigment epithelium (RPE) but not within the neural retina. Functionally, these CD19^+^ B cells isolated from the RPE can secrete IgM following LPS stimulation, with young WT cells producing more IgM per cell than aged WT cells, whereas μs−/− B cells lack secretion entirely. These results demonstrate that WT CD19^+^ B cells isolated from the RPE retain the capacity to respond to stimulation and secrete antibody. While stimulation of the total CD19^+^ B cell population found in the RPE demonstrated secretion of IgM, it is possible certain sub-populations of B cells within the total CD19^+^ B cells contribute more to secretion than others. Fractionation and subsequent analysis of sub-populations of B cells found within the RPE are important future directions.

Furthermore, single-cell V(D)J sequencing demonstrated age- and IgM-associated differences in the antibody repertoire of CD19^+^ B cells isolated from the RPE. Young WT RPE B cells predominantly utilize VH1, whereas young μs−/− B cells shift toward VH2 and VH5. Aging further diversifies the repertoire, with aged WT RPE B cells displaying increased utilization of VH11 and aged μs−/− B cells exhibiting increased VH2, VH6, and VH14 usage. Expansion of CDR-H3 replicates and junctional diversity also varied with age and secreted IgM availability, consistent with previous studies demonstrating age- and IgM-associated differences in antibody repertoires of other B cell populations in lymphoid tissues.^40^^,^^53^ Analysis of CDR-H3 loops revealed age- and IgM-associated alterations in hydrophobicity and amino acid composition, with aged μs−/− B cells showing increased hydrophobicity and aged WT B cells exhibiting a positive correlation between DH length and CDR-H3 hydrophobicity. These findings indicate that local IgM and aging may influence the biochemical and structural characteristics of retinal antibodies, which could impact antigen recognition, immune surveillance, and tissue homeostasis.

The total number of B cells typically declines with age due to reduced bone marrow output. Here, we also observed that aged WT and μs−/− mice demonstrated decreased numbers of B cells in the retinal pigment epithelium (RPE) as compared to young WT and μs−/− mice. The B220+CD19^+^CD5^−^CD43^−^phenotype we observed is consistent with B2-like cells described in CNS border regions, suggesting that the RPE might similarly harbor a locally maintained, non-circulating B cell population. Our results demonstrate the presence of B220+CD19^+^CD5^−^CD43^−^ B cells in both RPE and choroid/sclera; however, in the young their frequency is approximately 6-fold higher in the RPE. If B cells were derived from contaminating vasculature, one would expect enrichment in the highly vascular choroid rather than in the RPE. Consistent with this, our isolated RPE samples show no detectable expression of endothelial markers (vWF and CDH5), arguing against vascular carryover. Furthermore, if B cells detected in the RPE were derived from contaminating blood or vasculature, their phenotype might be expected to resemble circulating B cells. However, we found that RPE B cells display a distinct profile from B cells in circulation, including little to no CD23 expression with moderate CD21 levels and a marked absence of CD5^+^ B-1a cells, which are readily detected in circulation. In contrast to B cells found in the RPE, circulating B cells express significant levels of CD23 and CD21. While further experimentation is necessary, this difference in phenotype, together with the absence of endothelial markers and the enrichment of B cells in RPE relative to the highly vascular choroid, argues against a contamination-based explanation and supports a tissue-associated B cell population.

We found that RPE B cells exhibit age- and IgM-associated differences in their phenotype and activation. Young RPE B cells largely display a naive-like IgM+IgD+B220+CD19^+^CD43^−^phenotype with low expression of CD23, CD21, and PDL2, indicative of a homeostatic state. In the absence of secreted IgM, these cells upregulate CD80, consistent with activation and functional plasticity. We hypothesize this upregulation of CD80 is in response to barrier disruption and a pro-inflammatory cytokine milieu characterized by increased IL-6, IL-1β, TNFα, and VEGF, which we previously demonstrated occurs with age in the absence of IgM.^26^

These findings highlight the RPE as a site for CD19^+^ B cells, with a unique immune repertoire associated with aging and secreted IgM availability. Secreted IgM is critical for humoral immunity. Our previous study demonstrated that in the absence of IgM there is enhanced retinal degeneration during aging.^26^ This study demonstrates an association between IgM availability and differences in B cell activation, CDR-H3 diversity, and B-cell repertoire within the retinal barrier environment. The increased activation phenotype (CD80^+^) and emergence of IgG+ B cells in μs−/− mice suggest that loss of secreted IgM may prime RPE B cells toward a more inflammatory state, which could contribute to the age-related retinal dysfunction we previously published.^26^ These results raise the possibility that therapeutic modulation of IgM or targeted B cell interventions could preserve retinal homeostasis and potentially delay or mitigate retinal degenerative processes.

### Limitations of the study

These results collectively suggest an association between secreted IgM availability and the maintenance of retinal B cell homeostasis, ensuring both barrier integrity and a tolerogenic immune environment during aging. In the absence of IgM, we observe barrier disruption, cytokine shifts,^26^ and altered repertoire composition; however, our results do not establish direct causal relationships between these findings. The limitation of this study includes lack of functional consequences of the repertoire and CDR-H3 changes; as we did not assess antigen specificity, *in vivo* antibody function, or direct contributions to retinal immunity. Future studies should investigate how retinal B cells contribute to local immune regulation, whether altered repertoires influence antigen recognition or tissue homeostasis, and how secreted IgM modulates B cell selection and activation in barrier tissues. An additional important limitation of this study is the lack of direct visualization of B cells within intact RPE tissue. While multiple lines of evidence presented herein argue against vascular contamination, our data demonstrate B cells associated with the RPE but do not definitively establish residency within the RPE.

In conclusion, our study identifies a population of B cells isolated from the RPE, whose activation state, repertoire composition, and CDR-H3 properties are associated with both age and secreted IgM. These findings highlight the retina as a dynamic immune barrier tissue containing a distinct B cell population. Future studies are essential to determine how these B cells contribute to local immune surveillance, regulate retinal inflammation, and control susceptibility to age-related stress or pathology. Further investigation is also needed to ascertain whether these B cells are long-lived residents or represent a dynamically recruited population, as our current data do not distinguish between these possibilities. Additionally, understanding how RPE B cells interact with glial cells, endothelial barriers, and infiltrating immune cells under stress or disease conditions could uncover mechanisms of immune regulation at CNS-adjacent sites and may also leverage this B cell niche to prevent or treat retinal disease.

## Resource availability

### Lead contact

Requests for further information and resources should be directed to and will be fulfilled by the lead contact, Sarah E Webster (sarah.webster@wmed.edu).

### Materials availability

The study did not generate new unique reagents, and all mouse lines are available upon commercial request.

### Data and code availability

• Single cell VDJ sequencing data have been deposited in GenBank (NCBI BankIT) under the following accession numbers: PX769429-PX769911. The previously published bulk RNA-seq dataset reanalyzed in this study is available through NCBI Gene Expression Omnibus under accession number GSE156758 and was originally reported in Webster et al.^27^ All other datasets generated during and/or analyzed during the current study are available in Mendeley Data: Holodick, Nichol (2026), “Mouse RPE B Cell Data August 2026”, Mendeley Data, V1, https://doi.org/10.17632/rz5wyj3mxw.1.
• This paper does not include original code.
• Any additional information required to reanalyze the data reported in the paper is available from the lead contact upon request.

## Acknowledgments

The authors would like to thank the Flow Cytometry and Imaging Core in the Center for Immunobiology at the WMU Homer Stryker M.D. School of Medicine for support in the completion of the work. Figures were generated with BioRender.com under license: Holodick, N. (2026) https://BioRender.com/unh7ba1. Research reported in this publication was supported by the 10.13039/100000060National Institute of Allergy and Infectious Diseases (10.13039/100000060NIAID) of the 10.13039/100000002National Institutes of Health (10.13039/100000002NIH) under award 1R01AI154539-01 (N.E.H.) and F32AI174673-01A1 (S.E.W.). The content is solely the responsibility of the authors and does not necessarily represent the official views of the NIH.

## Author contributions

Conceptualization, S.E.W. and N.E.H.; methodology, S.E.W., N.E.H., and M.J.C.; investigation, S.E.W., N.E.H., M.J.C., N.L.T., D.H., and N.D.; visualization, S.E.W. and N.E.H.; supervision, N.E.H. and M.J.C.; writing – original draft, S.E.W.; writing – review and editing, S.E.W., M.J.C., and N.E.H.

## Declaration of interests

The authors declare no competing interests.

## STAR★Methods

### Key resources table

**Table:** 

REAGENT or RESOURCE	SOURCE	IDENTIFIER
**Antibodies**		

CD19	BD Biosciences	clone ID3; RRID: AB_3668953
CD43	BD Biosciences	clone s7; RRID: AB_2872201
B220/CD45	BD Biosciences	clone RA3-6B2; RRID: AB_2737894
CD23	BD Biosciences	clone B3B4; RRID: AB_3691121
CD5	BD Biosciences	clone 53-7.3; RRID: AB_3689285
CD80	BD Biosciences	clone 16-10A; RRID: AB_395039
PDL2	BD Biosciences	clone TY25; RRID: AB_3690083
CD73	BD Biosciences	clone TY/23; RRID: AB_10896329
IgM	BD Biosciences	clone R6-60.2; RRID: AB_2743732
IgD	BD Biosciences	clone 11.26c.2a; RRID: AB_10662544
IgG1	BD Biosciences	clone RM G1-1; RRID: AB_493293
CD21	BD Biosciences	clone 7G6l; RRID: AB_2739995
CD19-PE	BD Biosciences	clone ID3; RRID: AB_493995

**Biological samples**		

B6;*Ighm*^*tm1Che*^	Created by Dr. Jianzhu Chen and were kindly provided by Dr. Kishore Alugupalli	

**Chemicals, peptides, and recombinant proteins**		

DNA/RNA shield	Zymo	Cat# R1100
Aqua Live/Dead Stain	Invitrogen	Cat# L34965
collagenase I	Sigma	Cat# SCR103
collagenase XI	Sigma	Cat# C9407
DNAase I	Sigma	Cat# 10104159001
Hyaluronidase	Sigma	Cat# 37326-33-3
PBS, pH 7.4	Gibco	Cat# 10010023
HBSS	Gibco	Cat# 14170161
FBS	ThermoFisher	Cat# A5256801
MACS Buffer	Miltenyi	Cat# 130-091-221
RNAseOut	Invitrogen	Cat# 10777019
Cells-to-cDNA II Cell Lysis	Invitrogen	Cat# AM8723
Dithiothreitol	Thermo	Cat# R0861
IgePAL	Invitrogen	Cat# J61055.AP
lipopolysaccharide	eBioscience	cat# 00-4976-93
RPMI	Gibco	Cat# 11875093
L-glutamine	Thermo	Cat# 25030081
Penicillin-Streptomycin	Thermo	Cat# 10378016
2-mercaptoethanol	Gibco	Cat# 21985023
superscript III	Invitrogen	Cat# 18080044
random hexamers	Invitrogen	Cat# N8080127
ACK Lysis buffer	ThermoFisher	Cat# A1049201

**Critical commercial assays**		

anti-PE MicroBeads UltraPure	Miltenyi	Cat# 130-105-639
IgM ELISA	Stem Cell Technologies	Cat# 01999
Direct-zol RNA Miniprep Kit	Zymo	Cat# R2051
Qubit RNA HS Kit	Invitrogen	Cat# Q32855
RNA Pico Kit for Bioanalzyer	Agilent	Cat# 5067-1513
SuperScript III Platinum One-Step Kit with Rox	Thermo	Cat# 11745500

**Deposited data**		

Sequencing Data Deposited GeneBank	NCBI BankIT	PX769429-PX769911
Other Data	Mendeley Data	Holodick, Nichol (2026), “Mouse RPE B Cell Data August 2026”, Mendeley Data, V1, https://doi.org/10.17632/rz5wyj3mxw.1
Previously published neural retina bulk RNA sequencing dataset (Webster et al.27)	NCBI GEO	GSE156758

**Experimental models: Organisms/strains**		

Mouse: B6;*Ighm*^*tm1Che*^	Dr. Kishore Alugupalli	us
Mouse: B6;*Ighm*^*tm1Che*^	Dr. Kishore Alugupalli	WT (us+/+)
Mouse: C57BL/6J	Jackson Lab	000664

**Oligonucleotides**		

PrimeTime ActB	IDT	Mm.PT.58.22214843.g
PrimeTime B2M	IDT	Mm.PT.39a.22214835
PrimeTime Pmel	IDT	Mm.PT.58.32001339
PrimeTime RPE65	IDT	Mm.PT.58.45994766
PrimeTime vWF	IDT	Mm.PT.58.7361072
PrimeTime Cdh5	IDT	Mm.PT.58.7133590
MsV_H_E Primer	IDT	GGG AAT TCG AGG TGC AGC TGC AGG AGT CTG G
MsV_H_E Primer	IDT	ATG GCC ACC GAA TTC TTA TCA GA
MsV_H_N Primer	IDT	TGT AAA ACG ACG GCC AGT CAT TTG GGA AGG ACT GA

**Software and algorithms**		

FlowJo 10.10.0	BD Biosciences	
IMGT/HighV-Quest	Alamyar et al.^54^	
Prism 10.2.1	GraphPad	

### Experimental model and study participant details

#### In vivo animal studies

B6;129S4-*Ighm*^*tm1Che*^/J mice (referred to as “μs−/−” hereafter) are unable to produce the secreted form of IgM. The knock-out mice, originally created by Dr. Jianzhu Chen were kindly provided by Dr. Kishore Alugupalli. We backcrossed this line onto a pure C57BL/6J background within our facility, then bred and housed both WT and homozygous knockouts. Genome scanning was performed to confirm the percentage of C57BL/6J background after backcrossing was shown to be greater than 94%. C57BL/6J mice were also utilized (purchased from Jackson Laboratory and bred within our vivarium). Mice were housed at five mice per cage with a 12-h light/12-h dark cycle and *ad libitum* access to water and food. Mice were cared for and handled in accordance with the Guide for the Care and Use of Laboratory Animals (National Institutes of Health) and institutional guidelines. All animal studies were approved by the Western Michigan University Homer Stryker M.D. School of Medicine Institutional Animal Care and Use Committee under protocol number 2017-0003.

#### Sex as a biological variable

Both male and female mice were included in all experiments at 3–4 months of age (young) and 18–26 months of age (aged). Data from males and females were initially evaluated separately to assess potential sex-associated differences. No sex-associated differences were observed for the outcomes examined; therefore, data from male and female mice were combined for the analyses presented. Sample sizes for each experiment are indicated in the corresponding figure legends.

### Method details

#### Tissue preparations

Enucleations were performed on all euthanized mice. Retinal tissue was isolated as previously published.^26^^,^^55^^,^^56^^,^^57^ Briefly, mice were euthanized by CO2 asphyxiation followed by bilateral pneumothorax. Both eyes were removed, dipped in 70% alcohol and the cornea and lens were carefully removed from the eye to create an eyecup. The retina was then removed; the neural retina was excised first, and the RPE (dark brown) was isolated by dissecting it from the choroid via forceps as previously published.^58^ The excised RPE and choroid/sclera were then stored on ice-cold PBS for single cell suspensions or in DNA/RNAshield (Zymo) for RT-qPCR. All dissections were performed in parallel across genotypes and age groups to minimize batch effects. Peripheral blood was collected after euthanasia, and then red blood cells were lysed with ACK lysis buffer (Thermo Fisher). The cells were then washed and phenotyped using flow cytometry.

#### Retinal single cell suspensions

Excised neural retina, RPE, and choroid/sclera cells were dissociated to a single cell suspension using a modification of the protocol published by Weber et al. (2014) to maintain the viability of lymphocytes^59^ as traditional methods of retinal dissociations negatively impact B cells due to use of the papain enzyme.^60^^,^^61^^,^^62^^,^^63^ Briefly, the RPE and choroid/sclera tissue was subjected to enzymatic digestion with 450U/mL collagenase I, 125U/mL collagenase XI, 60U/mL DNase I, and 60U/mL hyaluronidase (Sigma) for 1h at 37C with shaking. The neural retina, RPE, and choroid/sclera suspension was then passed through a 70 μm filter. The single cell suspensions were used for either phenotyping or CD19^+^ B cell sorting. All dissociations were performed in parallel across genotypes and age groups to minimize batch effects.

#### Phenotyping B cells using flow cytometry

The RPE layer and choroid/sclera suspensions, or peripheral white blood cells, were resuspended in HBSS with 2.5% FBS, stained with immunofluorescent antibodies, and then analyzed on the Aurora CS flow cytometer (Cytek) with gating on live cells by forward side scatter and/or Aqua Live/Dead stain (Invitrogen). Images were constructed with FlowJo 10.10.0 software (BD Biosciences). The following antibodies were obtained from BD Biosciences: CD19 (clone ID3), CD43 (clone S7), B220/CD45 (clone RA3-6B2), CD23 (clone B3B4), CD5 (clone 53-7.3), CD80 (clone 16-10A), PDL2 (clone TY25), CD73 (clone TY/23), IgM (clone R6-60.2), IgD (clone 11.26c.2a), IgG1 (clone RM G1-1), and CD21 (clone 7G6). Single-cell stained compensation controls and fluorescence minus one (FMO) controls were used for gating. Gates were applied uniformly across all experimental groups.

#### CD19^+^ selection and cell sorting

The RPE layer and choroid/sclera suspensions were passed through a 40 μm filter and then stained with CD19-PE (clone ID3) in MACS buffer (1X PBS, 0.5% BSA, 2 mM EDTA) for 30 minutes on ice. CD19^+^ B cells were then positively selected from this single-cell suspension using the Miltenyi anti-PE MicroBeads UltraPure following manufacturer’s instructions. The positively selected B cells were spun down, resuspended in HBSS containing 2.5% FBS, and then sorted on an Influx cell sorter (BD Biosciences) with gating on live cells by forward side scatter and Aqua Live/Dead stain (Invitrogen). Images were constructed with FlowJo 10.10.0 software (BD Biosciences). The CD19 clone ID3 (BD Biosciences) antibody was used.

#### Single cell sequencing and analysis

RPE B cells were obtained from WT and μs−/− mice at the indicated age and stained with CD19-PE (clone ID3, BD Biosciences) and Aqua Live/Dead (Invitrogen). CD19^+^ B cells were single-cell sorted using an Influx cell sorter (BD Biosciences) into a 96-well plate containing lysis buffer (RNaseOut, 5X Buffer, DTT, IgePAL, carrier RNA, Invitrogen). Post-sort re-analysis of CD19^+^ B cells showed them to be ≥ 98% pure. To obtain cDNA, a 20μl reverse transcription reaction was run per well using the SuperScript III enzyme and random hexamers (Invitrogen). We then performed a semi-nested PCR reaction to amplify the VDJ region of the heavy chain as previously described.^64^^,^^65^ The PCR products were run on the Qiagen Qiaxcel. PCR products were sequenced (Genewiz) using the MsV_H_E primer. Sequences were analyzed using an online sequence analysis tool, IMGT/HighV-Quest. Only productive, in-frame heavy chain sequences were included in downstream analyses.

#### IgM ELISA analysis of B cell supernatants

RPE B cells were obtained from WT and μs−/− mice at the indicated age and stained with CD19-PE (clone ID3, BD Biosciences) and Aqua Live/Dead (Invitrogen). CD19^+^ B cells were bulk sorted using an Influx cell sorter (BD Biosciences). The cells were plated in a 96-well plate with 25 μg/ml of Lipopolysaccharide (LPS) in RPMI with 10% FBS, L-Glut, Pen/Strep, 2-ME for 72 hours at 37°C, 5% CO2. After 72 hours of culture with LPS, the cells were centrifuged and the supernatant collected. The supernatant was then evaluated for IgM by ELISA according to the manufacturer’s instructions (Stem Cell Technologies).

#### Gene expression analysis

For gene expression analysis, CD19^+^ B cells were bulk sorted from RPE or choroid/sclera preparations of male and female mice and stored in DNA/RNAshield. Because retinal B cells represent a rare cell population, sorted cells from multiple mice of the same sex and experimental group were pooled to obtain sufficient RNA. This procedure was performed in three independent experiments conducted on separate days, generating three biological replicates per sex and experimental group. Female and male samples were processed separately at all stages. The number of mice contributing to each pooled biological replicate varied by experiment and is indicated in the corresponding figure legends. RNA was isolated from pooled samples using the Direct-zol RNA Miniprep Kit (Zymo) following the manufacturer’s protocol. RNA concentration was measured using a Qubit 3, and RNA integrity was assessed using the RNA Pico kit on a Bioanalyzer (Agilent); only samples with RIN >8 were used for downstream analysis. PrimeTime Assays (IDT; TaqMan chemistry) were used for the genes listed in the key resources table, with assay efficiencies between 90–110%. RT-qPCR was performed using the SuperScript III Platinum One-Step Kit with ROX (Thermo Fisher Scientific), with 1 ng total RNA per 20 μL reaction. Each biological replicate was analyzed in technical triplicate. Reactions were run on a CFX Opus 96 Real-Time PCR System (Bio-Rad) using the following cycling conditions: 50 °C for 15 min for cDNA synthesis, 95 °C for 2 min for polymerase activation, followed by 40 cycles of 95 °C for 15 s and 60 °C for 30 s. Relative gene expression was calculated using the 2ˆΔCt method with ActB and B2M as endogenous reference genes, as previously published.^26^ No samples were excluded from analysis.

### Quantification and statistical analysis

Statistical analyses were performed using Prism (Version 10.2.1). Data distribution was assessed for normality prior to statistical testing, and appropriate parametric or nonparametric tests were applied as indicated in each figure legend. Statistics used: Sharpiro-Wilk test for normality, Mann-Whitney test, chi-square test, and Spearman’s rank correlation. All statistical analyses used are indicated in each figure legend. Sample sizes were determined based on prior studies. All analyses were reported with mean ± SEM with p<0.05 being considered statistically different. Asterisks for p values: ∗p<0.05, ∗∗p<0.01, ∗∗∗p<0.001, ∗∗∗∗p<0.0001.
