# Supplementary material for: B cells in the retinal pigment epithelium change with aging and IgM availability
Source: iScience. 2026 Sep 26;29(10):117640. doi: 10.1016/j.isci.2026.117640 (PMC13638799; doi:10.1016/j.isci.2026.117640)
Supplement: Supplementary file 1 [file mmc1.pdf]

iScience, Volume *29*

Supplemental information

B cells in the retinal pigment epithelium change with aging and IgM availability

Sarah E. Webster, Nicholas Deleon, Naomi L. Tsuji, Daken Heck, Michael J. Clemente, and Nichol E. Holodick

SUPPLEMENTAL INFORMATION

Supplemental Figure 1

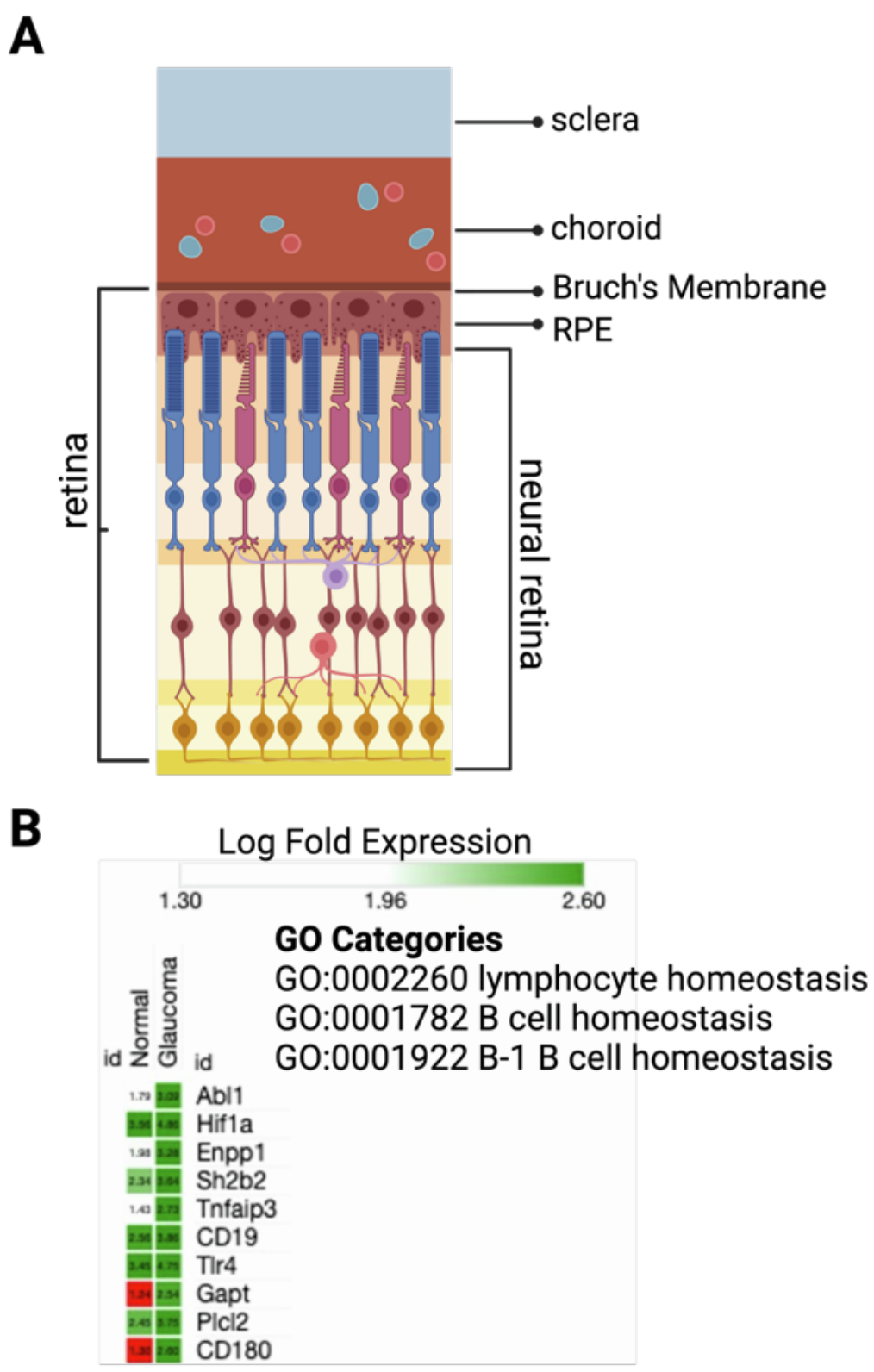

Supplemental Figure S1. Posterior eye anatomy and bulk RNA-sequencing of the neural retina. (A) A diagram of the posterior of the eye: the neural retina and retinal pigment epithelium compose the retina situated behind a membrane, separating the retina from the rest of the eye – the RPE compose a significant portion of the blood-retina-barrier. Posterior to the RPE is the choroid, the vascular layer of the posterior eye, covered by the most posterior structure, the sclera, a dense connective tissue layer. **(B)** Bulk RNA-seq data showing wild type neural retina as compared to a glaucomatous retina. CD19 expression is present in wild type retina, along with other genes related to B cell homeostasis. Previously published bulk RNA-seq data from the neural retina (NCBI GEO: GSE156758)[27] were reanalyzed for expression of B cell related genes.

Supplemental Figure 2

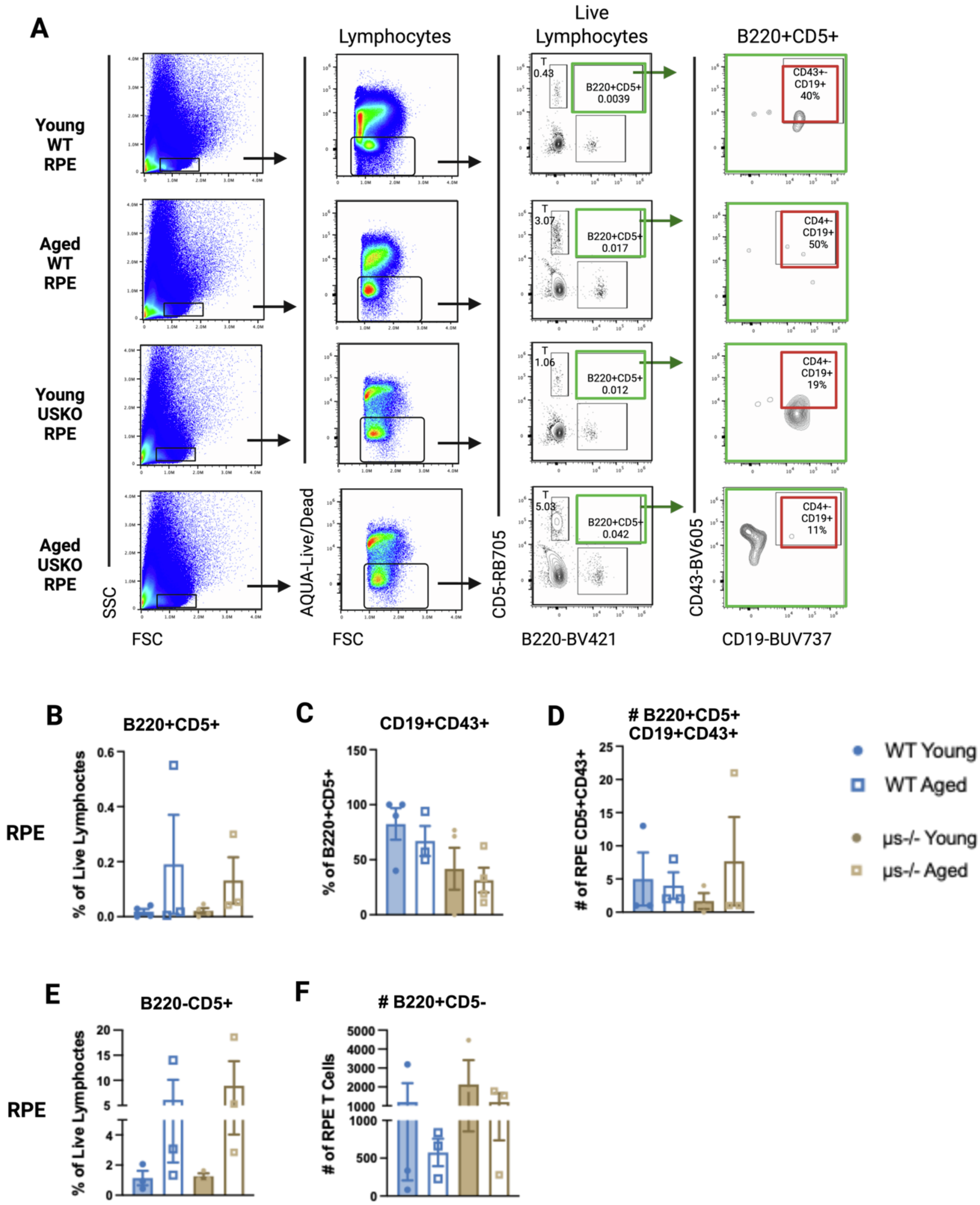

Supplemental Figure S2. Frequency of B220+CD5+ B cells found in the RPE. Murine RPE was isolated from young (3-4mo) and aged (22-24mo) WT and μs-/- mice from both male and female mice. The RPE was digested, prepared as a single cell suspension, and then stained for phenotype analysis. **(A)** Representative flow plots of B cell phenotyping from isolated RPE. **(B)** The percent of B220+CD5+ lymphocytes found in the RPE. **(C)** The percent of live lymphocytes in the RPE that are CD19+CD43+. **(D)** The number of B220+CD5+CD19+ CD43+ B cells found in the RPE. **(E)** The percent of live lymphocytes in the RPE that are B220-CD5+. **(F)** The number of B220+CD5- B cells found in the RPE. Results shown are an average of 3 independent experiments for young mice (individual animals pooled in each of 3 experiments with 3-6 mice per experiment) WT: n=17 mice/34 eyes, μs-/-: n=13 mice/26 eyes; and 3 independent experiments for aged mice (individual animals pooled in each of 3 experiments) WT: n=13 mice/26 eyes, μs-/: n=18 mice/36 eyes. Data are represented as mean +/- SEM. Statistics used: Sharprio-Wilk test for normality and Mann-Whitney test.

Supplemental Figure 3

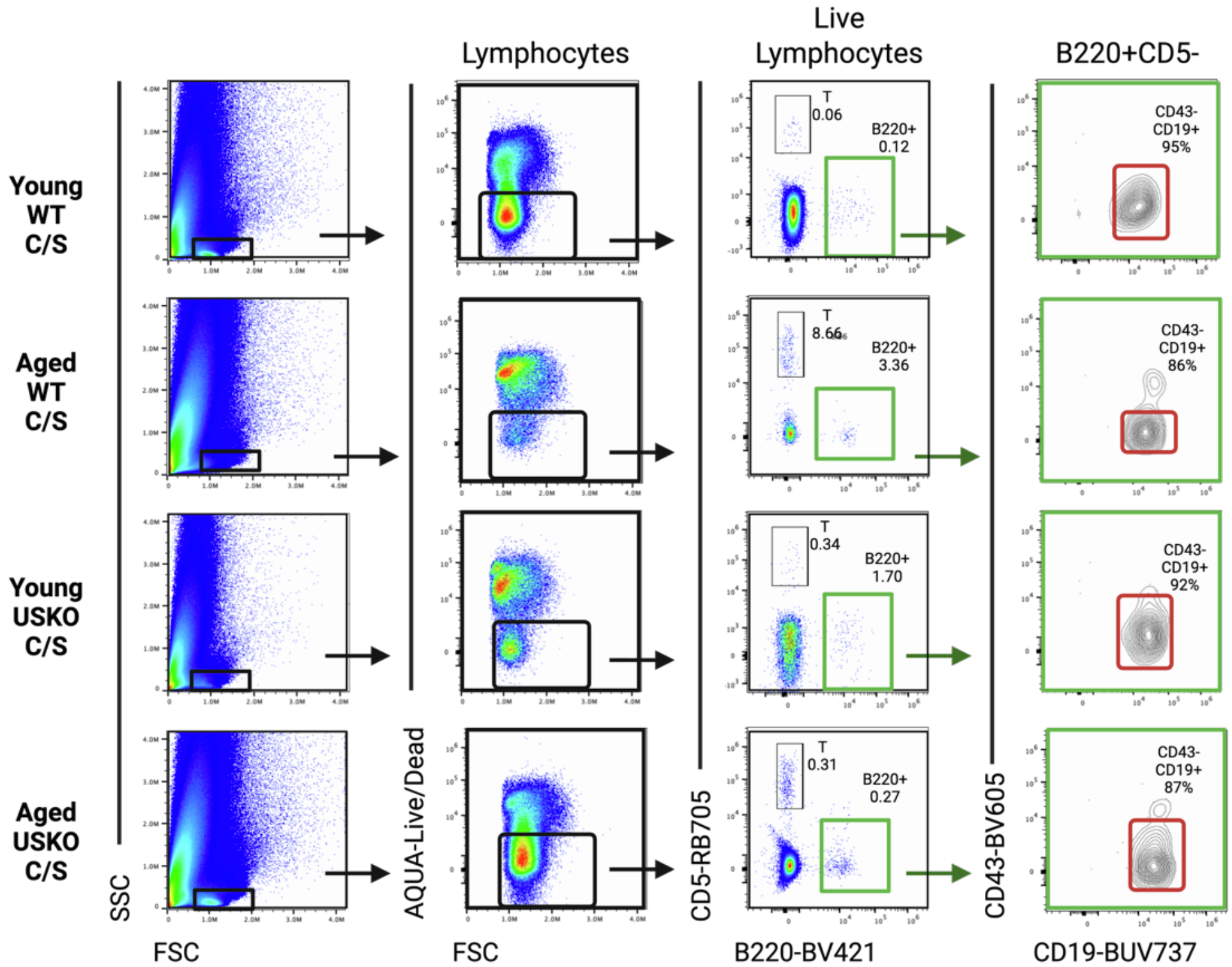

Supplemental Figure S3. Representative flow diagram of identification of B220+CD19+CD43-CD5- B cells from the c/s. Single cell suspensions were prepared from c/s tissue and processed for flow cytometry. Mice were grouped by age (young:3-4mo and aged:18-24mo) and background (WT and μs-/-). Results shown are an average of 3 independent experiments for young mice (individual animals pooled in each of 3 experiments with 3-6 mice per experiment) WT: n=17 mice/34 eyes, μs-/-: n=13 mice/26 eyes; and 3 independent experiments for aged mice (individual animals pooled in each of 3 experiments) WT: n=13 mice/26 eyes, μs-/-: n=18 mice/36 eyes. Data are represented as mean +/- SEM. Statistics used: Sharprio-Wilk test for normality and Mann-Whitney test.

Supplemental Figure 4

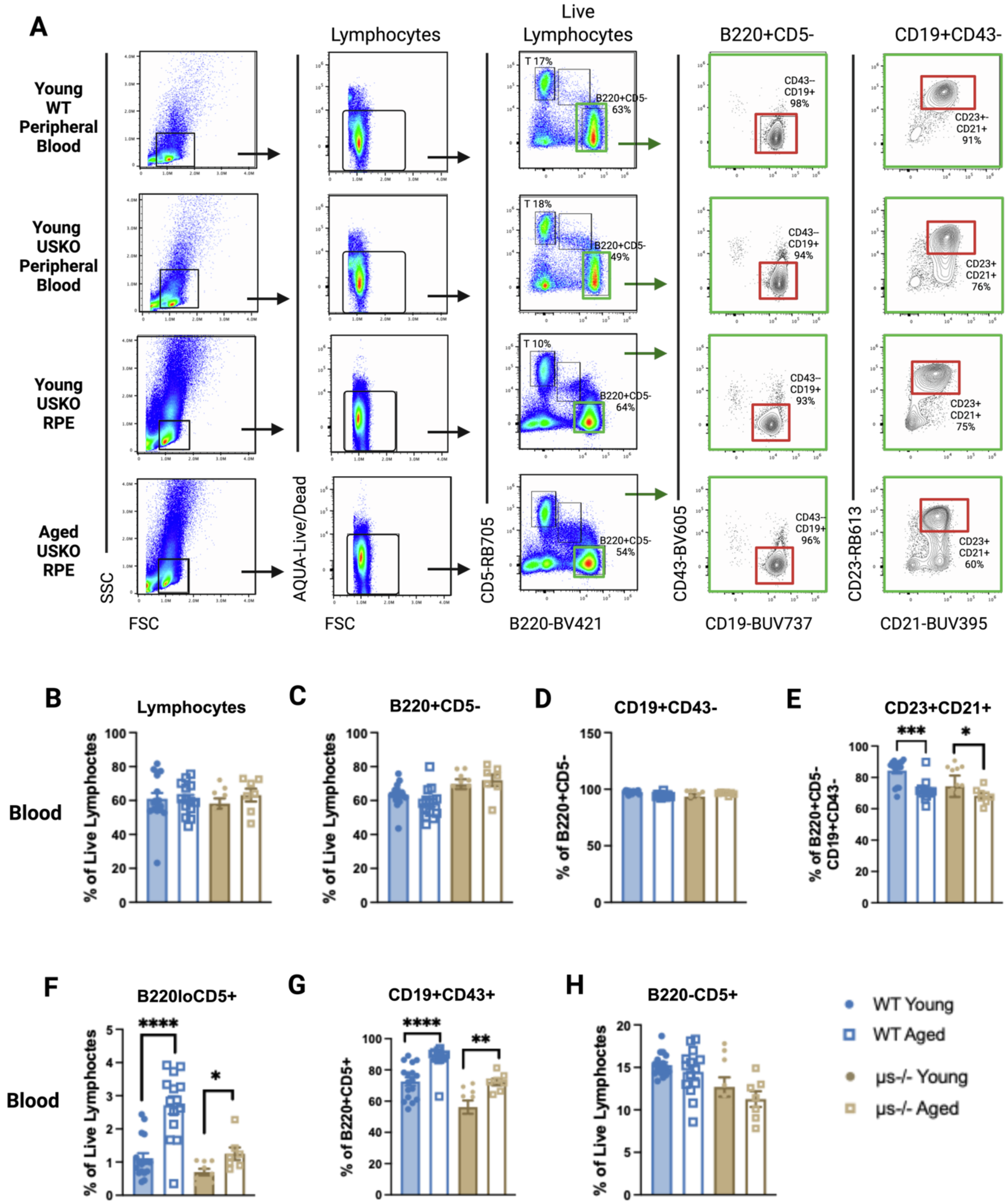

Supplemental Figure S4. Phenotype of B cells obtained from peripheral blood. Murine peripheral blood was isolated from young (3-4mo) and aged (22-24mo) WT and μs-/- mice from both male and female mice. The peripheral blood was exposed to RBC lysis buffer, washed, prepared as a single cell suspension, and then stained for phenotype analysis. **(A)** Representative flow plots of B cell phenotyping from peripheral blood white blood cells. **(B)** The percent of lymphocytes found in the peripheral blood (PB). **(C)** The percent of live lymphocytes in the PB that are B220+CD5-. **(D)** The percent of B220+CD5- B cells in the PB that are CD19+CD43-. **(E)** The percent of B220+CD5-CD19+CD43- cells in the PB that are CD23+CD21+. **(F)** The percent of live lymphocytes that are B220loCD5+ in the PB. **(G)** The percent of B220+CD5+ cells that are CD19+CD43+ in the PB. **(H)** The percent of live lymphocytes that are B220-CD5+ in the PB. Results shown are an average of 3 independent experiments. Young WT: n= 14 mice, young μs-/: n=19 mice; aged WT: n=11 mice, aged μs-/: n=7 mice. Data are represented as mean +/- SEM. Statistics used: Sharprio-Willk test for normality and Mann-Whitney test. Asterisks for p values: *p<0.05, **p<0.01, ***p<0.001, ****p<0.0001.
